# An Assessment of the Availability of Essential Medicines in Gadag Taluk, Karnataka: A Study of Primary Health Centers

**DOI:** 10.7759/cureus.39341

**Published:** 2023-05-22

**Authors:** S Tejesh, Nagaveni SJ

**Affiliations:** 1 School of Environmental Science, Public Health and Sanitation Management, Karnataka State Rural Development and Panchayat Raj University, Gadag, IND

**Keywords:** health system development, availability, universal health care for all, phc, essential medicines

## Abstract

Background

Essential medicines are those that meet the priority healthcare needs of most of the population and are included in the 2030 Agenda for Sustainable Development. The national list of essential medicines should be tailored to the specific needs of each nation and should always be accessible at reasonable prices and with guaranteed quality.

Methods

A cross-sectional study was done to assess the availability of essential medicines in primary health centers (PHCs) in Gadag Taluk. The data for the assessment of availability was collected using a checklist, which was prepared after reviewing the Karnataka list of essential medicines, surgical items, and miscellaneous items from 2021 to 2022 for PHCs. The sampling design was a universal sample of all 15 PHCs, as per health management information system data, to assess the availability of essential medicine in PHCs.

Results

Results indicate that the availability of essential medicines in 15 PHCs in Gadag Taluk is 74.20%. The availability of anti-allergic drugs and drugs used in anaphylaxis was around 88%, whereas the availability of antidiabetic drugs and non-steroidal anti-inflammatory drugs was 86.88% and 86.66%, respectively. All other categories of drugs are available above 50%, except ophthalmic and ear, nose, and throat drugs.

Conclusion

The public sector should be strengthened by making sure that patients have access to free essential medicines and that essential medications are always available. This would help patients spend less money out of their own pockets and move India closer to achieving universal healthcare.

## Introduction

Medicines are indispensable and necessary for the health needs of the population. They should always be available, in the proper dosage forms, to all segments of society, including the lowest level [1]. WHO has identified "essential medicines" as those that satisfy the priority healthcare needs of a population [1]. The necessity of having access to drugs is expressly acknowledged in the 2030 Agenda for Sustainable Development. Almost all of the health-related sustainable development goal three targets, including those pertaining to malaria, non-communicable diseases, maternity and child health, and sexual and reproductive health, depend on improved access to drugs. The significance of having access to medications and vaccinations is explicitly highlighted by targets 3.8 and 3.b [2]. In 1970, Tanzania was the first country in the world to build an essential medicine list. Following that, the World Health Assembly requested in 1975 that WHO support member nations in selecting and obtaining medicines while ensuring premium quality at competitive prices. About 186 drugs made up the initial WHO model list of essential medicines, which was later made public in 1977 [1,3].

Patients often assess a primary healthcare system's quality based on the qualified medical staff and accessibility to essential medications. A key element of the National Health Policy 2017 and universal healthcare is free access to needed medications. This is considered an important measure in the proposed National Health Assurance Mission of the government of India for 2014 [4]. Households in India are required to pay out-of-pocket expenses for medication, which is the second largest expense after food [5]. This is due to the limited availability of medicine in the public sector. In developing countries, up to 90% of the population pays for medication with out-of-pocket payments [6]. India is no exception, with 48.21% of the cost of healthcare being paid out-of-pocket and medication taking up 70% of that cost [7]. According to a study done in the union territory of South India, the availability of 50 essential drugs was found to be between 66% and 80% in the 10 public health institutions surveyed [8].

The provision of all essential medications at a fair price for the public is one of the key goals of Indian health policy [9]. The availability of medication is inconsistent in developing nations like India, particularly in public health facilities [10]. Surveys conducted across India revealed a shortage of necessary medications, particularly in public health facilities [11]. The availability of necessary medications has a direct impact on how well healthcare facilities operate [3]. The continuously shifting nature of the world economy and the scarcity of resources make the essential medicine concept more important than ever. The majority of healthcare requirements can be met with a small selection of well-chosen medications. The essential medicine concept is globally relevant and provides the most economical answer to healthcare requirements. This survey was conducted to assess the availability of essential medicines in primary health centers (PHCs) in Gadag Taluk, Karnataka. The findings of this survey could be utilized by healthcare decision-makers to comprehend the current state of accessibility to essential medicines in Gadag and recommend strategies to enhance availability, which would be to the advantage of patients.

## Materials and methods

Study design

A research study using a cross-sectional design was conducted to assess the availability of essential medicines in PHCs in Gadag Taluk.

Study duration and location

This research was conducted between November 2021 and March 2022 in Gadag Taluk, Gadag District, Karnataka.

Study sample size

The sampling design and size were universal, and all 15 PHCs in Gadag Taluk, as per the health management information system list of Karnataka, were selected.

Data collection tools

A comprehensive checklist of key medicines was developed following a review of the Karnataka list of essential medicines, surgical items, and miscellaneous items from 2021 to 2022 for PHCs. The Karnataka list of essential medicines for PHCs contains a total of 501 drugs, of which 104 are classified as essential, most essential, and very essential. These 104 drugs were employed to assess availability in the current study.

Data collection

A pilot study was conducted to ensure data collection was feasible. The list of medicines to be surveyed was kept private to not create any bias. On the day of the survey, the pharmacies of the health facilities were observed directly in the presence of pharmacists and medical officers to get data on the availability of essential medicines.

Data analysis

The quantitative data collected was processed with Microsoft Excel 2019 (Microsoft, Washington, USA) to generate frequencies, percentages, and graphs. The medicines were divided into therapeutic classes, and the percentage availability of a therapeutic class in a PHC was calculated by dividing the number of medicines available within that category in a PHC on the day of the survey by the total number of medicines within that category that should be available as per the list of medicines prepared. The percentage availability of individual medicines in all surveyed health facilities (number of PHCs in which individual medicines were available/total number of PHCs) was also calculated. A drug was thought of as accessible if it was in the inventory on the day of the research.

Ethical clearance

The Karnataka State Rural Development and Panchayat Raj University Institutional Ethical Committee (RDPRU/SEP/IEC/3/2021) gave its approval to this work. The District Health and Family Welfare office (2021-22/494) of the Gadag District was also consulted regarding administrative authorization for data collection from state government public health facilities.

## Results

The availability of essential medicines in 15 PHCs in Gadag Taluk is 74.20%. The availability of antiallergic drugs and drugs used in anaphylaxis was around 88%, whereas the availability of antidiabetic drugs and non-steroidal anti-inflammatory drugs (NSAIDs) was 86.88% and 86.66%, respectively. All other categories of drugs are available above 50%, except ophthalmic and ear, nose, and throat (ENT) drugs. The percentage of medicines available by therapeutic category in PHCs in Gadag Taluk is given in Table 1.

**Table 1:** Availability of medicines (%) by therapeutic category in PHCs of Gadag Taluk n = number of PHCs NSAIDs: non-steroidal anti-inflammatory drugs, ENT: eyes, nose, and throat

Sl. No	Essential drug categories	% availability (n=15)
1	NSAIDs	86.66
2	Antibiotics	73.33
3	Anti-allergic and drugs used in anaphylaxis	88.00
4	Bronchodilators	71.43
5	Gastro-intestinal, anti-ulcer drugs, and intestinal anthelmintics	84.44
6	Cardiovascular drugs	61.11
7	Anti-diabetic drugs	86.88
8	Obstetrics drugs	69.44
9	Tranquilizers, sedatives, psychiatric drugs	59.16
10	Anesthetics	83.33
11	Anti-dotes, vaccines, and sera	65.56
12	IV infusions and other electrolytes	76.67
13	Dermatological, ophthalmic, and ENT drugs	40.00
14	Vitamins and haematinics	71.79
15	Sterile and disposable items	78.10
	Total	74.20

**Figure 1:**
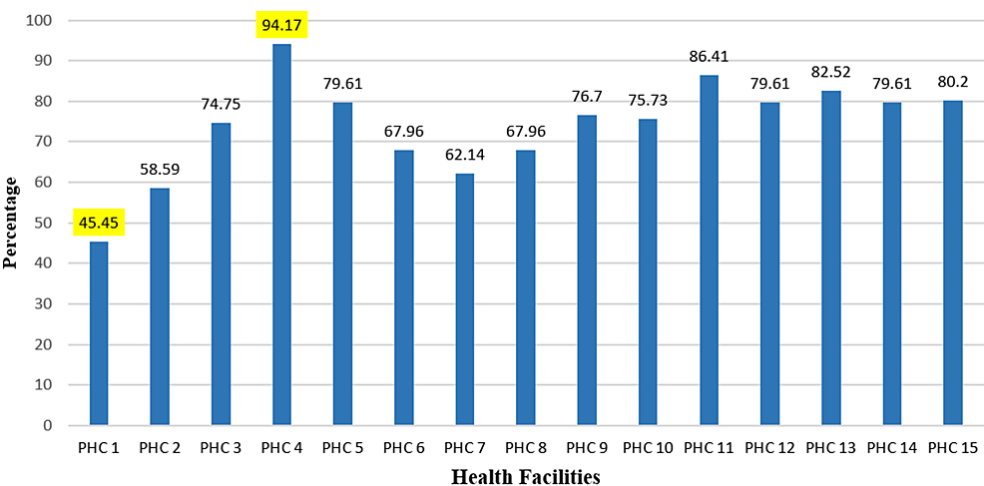
Percentage availability of essential medicines in Gadag Taluk PHC (facility)-wise PHC: primary health center

The findings of this study demonstrate that essential medicines have a wide availability range across the PHCs of Gadag Taluk. PHC 4 has the highest availability rate of 94.17%, while PHC 1 has the lowest availability rate of 45.45%. These results show that the availability of essential medicines is highly dependent on the PHC and highlight the importance of increasing access to these essential medicines in areas of low availability. The percentage availability of essential medicines in Gadag Taluk PHC (facility)-wise is shown in Figure 1.

Out of the 104 medicines assessed, glyceryl trinitrate and anti-D immunoglobin are two essential medications that are unfortunately not available in any PHC. This is extremely concerning, as these medications are essential to treating certain medical conditions. This lack of availability could be potentially life-threatening for many individuals, and it is imperative that steps are taken to ensure these medications are made more accessible. An in-depth breakdown of each medication's availability by percentage in the PHCs surveyed is given in Table 2.

**Table 2:** An in-depth breakdown of each medication's availability by percentage in the PHCs surveyed n = PHCs DPT: diphtheria, pertussis, and tetanus

Sl. No	Essential drug name	Strength	% availability (n=15)
1	Absorbent cotton wool	500gm	93.33
2	Adrenaline bitartrate	1mg/ml injection	66.67
3	Albendazole	400mg tab	100.00
4	Ambroxol	30mg/5ml syrup	80.00
5	Amikacin	100mg/2ml syrup	73.33
6	Amitriptyline	25mg tablet	66.67
7	Amlodipine	5mg tablet	100.00
8	Amoxicillin + clavulanic acid	200mg +28.5mg/5ml powder	73.33
9	Amoxycillin	250mg/5ml syrup	80.00
10	Amoxycillin	125mg/5ml powder	86.67
11	Amoxycillin + clavulanic acid	625mg tablet	60.00
12	Anti-D immunoglobin	300mcg injection	0.00
13	Antisnake venom	NA	93.33
14	Atenolol	50mg tablet	93.33
15	Atropine sulfate	1mg/ml injection	53.33
16	Calcium + vit D	500 mg	93.33
17	Calcium gluconate	10 % W/V/ 10ml Inj	13.33
18	Cefixime	50mg/5ml syrup	60.00
19	Chlorpheniramine maleate	4mg tablet	100.00
20	Chlortalidone	12.5mg tablet	33.33
21	Ciprofloxacin hydrochloride	500mg tablet	100.00
22	Ciprofloxacin hydrochloride eye/ear	0.3%w/v drops	33.33
23	Co-trimoxazole (trimethoprim + sulfamethoxazole)	40+200mg/5ml syrup	73.33
24	Dexamethasone	4mg/ml injection	93.33
25	Dextrose	5% injection	66.67
26	Dextrose with sodium chloride	5% + 0.9% infusion	80.00
27	Diclofenac	25mg/ml injection	100.00
28	Diclofenac	50mg tablet	100.00
29	Diclofenac	75mg/ml injection	66.67
30	Dicyclomine hydrochloride	10mg/ml injection	93.33
31	Dicyclomine hydrochloride	10mg tablet	100.00
32	Disposable mask	NA	100.00
33	Domperidone	1mg/ml syrup	53.33
34	Domperidone	10mg tablet	93.33
35	DPT vaccine	0.5ml injection	93.33
36	Fluoxetine hydrochloride	60mg capsules	33.33
37	Folic acid tabs	500 mcg	100.00
38	Glibenclamide	5 mg tablet	93.33
39	Glimepiride	2mg tablet	93.33
40	Glimepiride	1mg tablet	93.33
41	Glyceryl trinitrate	0.5mg sublingual	0.00
42	Hydrocortisone sodium succinate	100mg injection	86.67
43	Ibuprofen	200mg tablet	93.33
44	Ibuprofen	400mg tablet	46.67
45	Iron-folic acid tab	100mg/500mcg	100.00
46	Iron-folic acid tab	20mg/100mcg	73.33
47	Iron sucrose	100 mg/5ml Inj	80.00
48	Levocetirizine	5mg tablet	66.67
49	Lignocaine inj	2 % w/v	83.33
50	Magnesium sulfate	500mg/ml injection	33.33
51	Metformin	1000mg tablet	100.00
52	Metformin	500mg tablet	100.00
53	Metronidazole	200mg/5ml syrup	6.67
54	Metronidazole	400mg tablet	100.00
55	Ondansetron	2mg/ml injection	46.67
56	Oral rehydration salts	WHO standard	100.00
57	Oxytocin	5IU/ml injection	60.00
58	Pantoprazole	40mg tablet	93.33
59	Paracetamol	125mg/5ml syrup	100.00
60	Paracetamol	500mg tablet	100.00
61	Pheniramine maleate	22.75 mg/ml injection	93.33
62	Phenobarbitone	30mg tablet	86.67
63	Phenobarbitone	60mg tablet	60.00
64	Phenytoin sodium	100mg tablet	93.33
65	Povidone iodine ointment	5 W/W	93.33
66	Pralidoxime chloride	1gm	66.67
67	Premix insulin 30:70	40IU/ml injection	46.67
68	Rabies vaccine	2.5IU injection	86.67
69	Ranitidine	25mg/ml injection	80.00
70	Ranitidine	150mg tablet	100.00
71	Ringer lactate	Injection	93.33
72	Risperidone	2mg tablet	93.33
73	Risperidone	4mg tablet	6.67
74	Salbutamol	5mg/ml inhalation	80.00
75	Salbutamol sulfate	2mg/5ml syrup	73.33
76	Salbutamol sulfate	4mg tablet	46.67
77	Silver sulfadiazine	125g cream	46.67
78	Sodium chloride 100ml	0.9%w/v	33.33
79	Sodium chloride 500ml	0.9%w/v	86.67
80	Sterile blood lancets	NA	80.00
81	Sterile disposable IV cannula with inj port	18G	53.33
82	Sterile disposable IV cannula with inj port	22G	80.00
83	Sterile disposable IV cannula with inj port	20G	46.67
84	Sterile disposable perfusion (infusion) sets with filter	NA	66.67
85	Sterile disposable syringe with hypodermic needle	2ml-23G syringe	100.00
86	Sterile disposable syringe with hypodermic needle	5ml-23G syringe	93.33
87	Sterile disposable umbilical cord clamp	NA	100.00
88	Sterile surgical gloves (latex)	7.5in	46.67
89	Sterile surgical gloves (latex)	6.5in	93.33
90	Sterile surgical gloves (latex)	7in	100.00
91	Sterile surgical gloves (latex)	6in	46.67
92	Telmisartan	40mg tablet	73.33
93	Theophylline + etophylline	50.6mg+169.4mg/2ml injection	93.33
94	Theophylline + etophylline	23mg+77mg syrup	33.33
95	Theophylline + etophylline	23mg+77mg tablet	93.33
96	Trihexyphenidyl hydrochloride	2mg tablet	33.33
97	Triple layer mask	NA	93.33
98	Vitamin A capsules	25000 IU	13.33
99	Vitamin A solution	1 lakh IU/ml 100 ml bottle	80.00
100	Vitamin C chewable tab	500 ml tab	93.33
101	Vitamin K	10 mg/ml Inj	53.33
102	Zinc drops	20mg/ml drops	93.33
103	Zinc sulfate	100 mg tab	73.33
104	Zinc syrup	60 ml	66.67
105	Total		74.20

## Discussion

The availability of medications at each facility is an essential performance criterion [12]. If necessary medications are not easily accessible, patients may be deterred from visiting medical institutions or may even cease using the healthcare system altogether [12]. This study has provided a glimpse of the availability of essential medicines in the PHCs of Gadag Taluk, with results indicating an overall median availability of 74.20%, slightly lower than the 80% benchmark set by WHO and Health Action International [13,14]. There could be several factors contributing to the insufficient availability of medications in the public sector, such as inadequate funding, the purchase of unnecessary medications, inaccurate forecasting of demands, and inefficient management of the drug supply chain [14].

This research's outcomes are consistent with those reported in a different study that was done in South India, wherein the availability of the essential medicines that were looked into in the public sector was seen to be 76% [8]. This study's findings are higher than the findings of other similar studies conducted in Odisha, five Indian states, Delhi, Chhattisgarh, Punjab, and Haryana, where the availability of surveyed essential medicines in the public sector was found to be 17% in Odisha [15], 41.3% in Delhi [16], 64.5% in Chhattisgarh, 45.2% in Punjab, and 51.1% in Haryana [17], respectively. The findings of this study are lower than those of other studies conducted in Shivmoga [18] and Andhra Pradesh [19], both of which reported the availability of surveyed essential medicines in the public sector to be 82% and 100%, respectively. All of these previous studies only looked at a selected basket of drugs, and until now, there have been no studies conducted in India or Karnataka that have assessed the complete essential medicine list. This study is the first of its kind to do so.

The investigation done in this study revealed that the overall availability of NSAIDs was 87.7%, which is higher than the other studies carried out in Haryana and Punjab [17]. Additionally, the availability of a selected number of drugs within the NSAID category was found to be higher compared to other drugs in the same group, possibly due to a better supply of the selected medications and the prescription patterns of physicians. The availability of affordable, high-quality antibiotics is a major global issue. Without these medications, the risks of surgery and the management of communicable diseases rise. It is critical for the prevention and treatment of bacterial infections, yet excessive and inappropriate use must be discouraged in order to avoid antibiotic resistance. According to the present investigation, the average availability of antibiotics is 74.7%, which is higher than the results from other research studies conducted in Haryana, Punjab, and 20 low- and middle-income countries, with percentages of 59.1%, 50.8%, and 48.9%, respectively [17,20].

The present study revealed that the availability of antiallergic medicines was 88%, which is higher than the 49.6% and 59.7% availability of essential medicines in the public sector in Punjab and Haryana, respectively [17]. This indicates good utilization and supply from the warehouse. Similarly, the availability of cardiovascular drugs was found to be 71.43%, which is comparable to the 70% availability of essential medicines in a Sri Lankan district [21]. The findings of the current research reveal that the supply of antidotes, vaccines, and serum is at 65.56%, which is below the standard set by the WHO [13,14], which may be because of a shortage of supply from the warehouse. When specific antidotes are not available, the life of a patient is in jeopardy. Thus, it is essential to ensure that a continuous supply of essential drugs is available in order to provide three levels of care to seriously ill patients.

A key drawback of this research was that the quantitative results were only collected on the day of the survey, potentially not providing an accurate representation of the monthly or yearly availability of the medicines.

## Conclusions

The present study aimed to assess the availability of essential medicines in PHCs in Gadag Taluk. The findings of the study revealed that the availability of essential medicines in PHCs in Gadag Taluk was 74.20%. This research provides valuable insight into the situation of essential medicine availability in Gadag Taluk. The primary conclusion of this research is that although the availability of essential medicines in PHCs in Gadag Taluk is relatively high, there are still gaps in the supply of essential medicines. This indicates that there is a need to further improve the availability of essential medicines in order to ensure access to quality healthcare for all. The key takeaways from this research are that there is a need to further invest in the health infrastructure of Gadag Taluk to ensure that all PHCs have adequate supplies of essential medicines. Furthermore, it is important to create awareness among the people of Gadag Taluk about the availability of essential medicines and their importance in providing quality healthcare.

## Appendices

The list of essential medicines used in the survey has been listed in Table 3.

**Table 3:** Karnataka list of essential medicines, surgical, and miscellaneous items from 2021 to 2022 for PHCs The list is available on the Mahitikanaja Karnataka government website.

Drug code	Drug name	Strength	Dosage	PHC
6.3.2	Albendazole	400mg	Tablet	Essential drug List
19.1.1	Ambroxol	30mg/5ml	Syrup	Essential drug List
6.7.1	Amikacin	100mg/2ml	Injection	Essential drug List
6.6.42	Amoxycillin	250mg/5ml	Oral Liquid	Essential drug List
6.6.44	Cefixime	50mg/5ml	Powder	Essential drug List
1.3.6	Diclofenac	25mg/ml	Injection	Essential drug List
1.3.7	Diclofenac	50mg	Tablet	Essential drug List
28.23.9	Disposable mask triple layer elastic type	NA	NA	Essential drug List
16.3.1	Domperidone	1mg/ml	Syrup	Essential drug List
16.3.2	Domperidone	10mg	Tablet	Essential drug List
20.1.25	Folic Acid	400mcg	Tablet	Essential drug List
1.3.10	Ibuprofen	200mg	Tablet	Essential drug List
25.3.6	Imipramine	25mg	Tablet	Essential drug List
20.1.24	Iron and folic acid tablets large (each sugar-coated tablet) contains dried ferrous sulfate IP (Eq to 60 mg of elemental iron and folic acid 500mcg (sugar-coated and red color)	60mg + 500mcg	Tablet	Essential drug List
25.2.2	Lorazepam	1mg	Tablet	Essential drug List
25.2.3	Lorazepam	2mg	Tablet	Essential drug List
16.1.10	Pantoprazole	40mg	Tablet	Essential drug List
4.1.10	Phenobarbitone	60mg	Tablet	Essential drug List
4.1.9	Phenobarbitone	30mg	Tablet	Essential drug List
4.1.14	Phenytoin sodium	100mg	Tablet	Essential drug List
12.1.5	Povidone iodine	null	Ointment	Essential drug List
25.4.14	Risperidone	2mg	Tablet	Essential drug List
25.4.15	Risperidone	4mg	Tablet	Essential drug List
12.1.10	Silver sulfadiazine	125g	Cream	Essential drug List
28.2.3	Sterile disposable IV cannula with inj port	18G	NA	Essential drug List
28.2.4	Sterile disposable IV cannula with inj port	20G	NA	Essential drug List
28.2.5	Sterile disposable IV cannula with inj port	22G	NA	Essential drug List
28.11.1	Sterile surgical gloves (latex)	null	NA	Essential drug List
28.11.2	Sterile surgical gloves (latex)	null	NA	Essential drug List
28.11.4	Sterile surgical gloves (latex)	null	NA	Essential drug List
9.1.6	Trihexyphenidyl hydrochloride	2mg	Tablet	Essential drug List
27.1.32	Vitamin A	100000 IU	Capsule	Essential drug List
27.1.30	Vitamin C chewable (ascorbic acid)	500mg	Tablet	Essential drug List
16.2.7	Zinc sulfate	20mg/ml	Drops	Essential drug List
16.2.8	Zinc sulfate	20mg/5ml	Syrup	Essential drug List
28.5.1	Absorbent cotton wool	500 GM	NA	Most essential drug list
3.1.1	Adrenaline bitartrate	1mg/ml	Injection	Most essential drug list
6.6.7	Amoxicillin + clavulanic acid	200mg + 28.5mg/5ml	Powder for suspension	Most essential drug list
6.6.8	Amoxicillin + clavulanic acid	625mg	Tablet	Most essential drug list
6.6.39	Amoxycillin powder	125mg/5ml	Suspension	Most essential drug list
18.2.1	Anti-D immunoglobin (human)	300mcg	Injection	Most essential drug list
5.2.1	Antisnake venom (polyvalent lyophilized)	NA	Powder for Infusion	Most essential drug list
11.3.6	Atenolol	50mg	Tablet	Most essential drug list
5.2.2	Atropine sulfate	1mg/ml	Injection	Most essential drug list
27.1.8	Calcium carbonate with vitamin D3	500MG+250IU	Tablet	Most essential drug list
6.7.14	Ciprofloxacin hydrochloride	500mg	Tablet	Most essential drug list
22.2.3	Ciprofloxacin hydrochloride eye/ear	0.3%w/v	Drops	Most essential drug list
3.1.5	Dexamethasone	4mg/ml	Injection	Most essential drug list
26.2.1	Dextrose	null	Injection	Most essential drug list
26.2.4	Dextrose with sodium chloride	5% + 0.9%	Infusion	Most essential drug list
1.3.33	Diclofenac	75mg/ml	Injection	Most essential drug list
16.5.2	Dicyclomine hydrochloride	10mg/ml	Injection	Most essential drug list
18.4.8	Diptheria and tetanus vaccine .5ML	NA	Vaccine	Most essential drug list
20.1.29	Ferrous salts (A) + folic acid (B) 20mg elemental iron (A) + 100mcg (B)	20 mg + 100 mcg IP/BP/USP	Syrup	Most essential drug list
17.7.6	Glimepiride	2mg	Tablet	Most essential drug list
11.1.7	Glyceryl trinitrate (sublingual)	0.5mg	Tablet	Most essential drug list
17.1.3	Hydrocortisone sodium succinate	100mg	Injection	Most essential drug list
20.1.9	Iron sucrose	100mg/5ml	Injection	Most essential drug list
3.1.22	Levo cetrizine	5mg	Tablet	Most essential drug list
2.2.20	Lignocaine hydrochloride (1x30ml)	2% w/v	Injection	Most essential drug list
4.1.6	Magnesium sulfate	500mg/ml	Injection	Most essential drug list
6.7.29	Metronidazole	200mg/5ml	Syrup	Most essential drug list
6.7.31	Metronidazole	400mg	Tablet	Most essential drug list
16.3.6	Ondansetron	2mg/ml	Injection	Most essential drug list
26.1.2	Oral rehydration salts	Sodium chloride 2.6, glucose anhydrous 13.5, potassium chloride 1.5, trisodium citrate dihydrate 2.9	Powder for solution	Most essential drug list
23.2.11	Oxytocin	5IU/ml	Injection	Most essential drug list
1.3.18	Paracetamol	125mg/5ml	Syrup	Most essential drug list
1.3.19	Paracetamol	500mg	Tablet	Most essential drug list
3.1.10	Pheniramine maleate	22.75 mg/ml	Injection	Most essential drug list
5.2.11	Pralidoxime chloride (2- PAM)	1g	Injection	Most essential drug list
18.3.1	Rabies vaccine	2.5IU	Injection	Most essential drug list
16.1.11	Ranitidine	25mg/ml	Injection	Most essential drug list
26.2.9	Ringer lactate	As per IP	Infusion FFS	Most essential drug list
19.1.12	Salbutamol nebuliser	5mg/ml	Solution	Most essential drug list
26.2.12	Sodium chloride (normal saline)	0.9%w/v	Injection FFS	Most essential drug list
28.12.1	Sterile disposable perfusion (infusion) sets with filter	Adult	NA	Most essential drug list
28.25.3	Sterile disposable syringe with hypodermic needle	2ml-23G	NA	Most essential drug list
28.25.5	Sterile disposable syringe with hypodermic needle	5ml-23G	NA	Most essential drug list
28.23.4	Sterile disposable umbilical cord clamp	NA	NA	Most essential drug list
28.11.3	Sterile surgical gloves (latex)	null	NA	Most essential drug list
27.1.25	Vitamin A pediatric (concentrated)	1,00,000IU/ml	Solution	Most essential drug list
20.2.18	Vitamin K IV	1mg/0.5ml	Injection	Most essential drug list
16.2.5	Zinc sulfate	20mg	Dispersible Tablet	Most essential drug list
25.3.1	Amitriptyline	25mg	Tablet	Very essential drug list
11.3.3	Amlodipine	5mg	Tablet	Very essential drug list
27.1.9	Calcium gluconate	100mg/ml	Injection	Very essential drug list
3.1.4	Chlorpheniramine maleate	4mg	Tablet	Very essential drug list
11.3.8	Chlorthalidone	12.5mg	Tablet	Very essential drug list
6.7.15	Co-trimoxazole (trimethoprim + sulfamethoxazole)	40+200mg/5ml	Suspension	Very essential drug list
16.5.4	Dicyclomine hydrochloride	10mg	Tablet	Very essential drug list
25.3.9	Fluoxetine hydrochloride	60mg	Capsule	Very essential drug list
17.7.5	Glimepiride	1mg	Tablet	Very essential drug list
1.3.11	Ibuprofen	400mg	Tablet	Very essential drug list
17.7.36	Metformin SR	500mg IP/BP/USP	Tablet	Very essential drug list
17.7.37	Metformin SR	1000 mg IP/BP/USP	Tablet	Very essential drug list
17.7.12	Premix insulin 30:70 1x10ml	40IU/ml	Injection	Very essential drug list
16.1.12	Ranitidine	150mg	Tablet	Very essential drug list
19.1.14	Salbutamol sulfate	2mg/5ml	Syrup	Very essential drug list
19.1.16	Salbutamol sulfate	4mg	Tablet	Very essential drug list
11.3.28	Telmisartan	40mg	Tablet	Very essential drug list
19.1.19	Theophylline + etophylline	50.6mg+169.4mg/2ml	Injection	Very essential drug list
19.1.20	Theophylline + etophylline	23mg+77mg	Syrup	Very essential drug list
19.1.21	Theophylline + etophylline	23mg+77mg	Tablet	Very essential drug list
